# Relationship between the expression of copper death promoting factor SLC31A1 in papillary thyroid carcinoma and clinicopathological indicators and prognosis

**DOI:** 10.1515/med-2025-1220

**Published:** 2025-10-28

**Authors:** Chenkun He, Rongrong Liu, Tianli Zhou

**Affiliations:** Department of Nuclear Medicine, Hunan Provincial People’s Hospital, No. 61 Jiefang West Road, Changsha, Hunan, 410005, P.R. China; Department of Nuclear Medicine, Hunan Provincial People’s Hospital, Hunan, 410005, P.R. China

**Keywords:** papillary thyroid carcinoma, SLC31A1, clinicopathological index, prognosis, Cox regression analysis, predictive value

## Abstract

**Objective:**

This study investigates the association between serum SLC31A1, a copper death-promoting factor, and clinicopathological features/prognosis in papillary thyroid carcinoma (PTC).

**Methods:**

A cohort of 250 PTC patients was stratified into good (*n* = 205) and poor (*n* = 45) prognosis groups. Clinicopathological parameters (age, sex, tumor diameter, differentiation, TNM stage, thyroid exocapsular invasion, lymph node metastasis) and serum SLC31A1 levels (measured via ELISA) were analyzed. Patients were further categorized into high/low SLC31A1 expression groups based on median values. Prognostic correlations were evaluated using Kaplan–Meier survival analysis, Cox regression, and receiver operating characteristic (ROC) curve assessment.

**Results:**

Elevated serum SLC31A1 levels were significantly associated with poor prognosis, advanced TNM stages (III/IV), poor differentiation, thyroid exocapsular invasion, and lymph node metastasis. Multivariate Cox analysis identified high SLC31A1 as an independent predictor of poor prognosis (HR = 1.235, 95% CI 1.158–1.317). ROC analysis demonstrated strong predictive accuracy for SLC31A1 in prognosis assessment (AUC = 0.859).

**Conclusion:**

High serum SLC31A1 expression correlates with aggressive clinicopathological features and independently predicts adverse outcomes in PTC patients, suggesting its potential as a prognostic biomarker for clinical stratification.

## 1 Introduction

Standing as the most frequently diagnosed thyroid malignancies, the incidence of papillary thyroid carcinoma (PTC) is climbing globally at an annual increase rate of 6% [1]. While PTC generally has a good prognosis, relapse and metastasis still occur in some patients [2,3], so finding new biomarkers as well as therapeutic targets is crucial to improve patient prognosis. Advances in molecular biology have led researchers to explore further mechanisms about the metastasis of PTC, aiming to uncover new diagnostic indicators and therapeutic approaches.

As a trace element necessary for body, copper plays a critical role in various biological functions, including antioxidant defense, iron homeostasis, and cell respiration [4]. However, abnormal intracellular copper levels have been linked to multiple pathological conditions, especially cancer [5,6]. Copper death (Cuproptosis) is a newly identified novel form of cell death, which leads to an increase in intracellular oxidative stress through the accumulation of copper ions, ultimately triggering cell death [7]. This newly discovered cell death mechanism has shown promising implications in cancer biology, suggesting potential therapeutic applications in oncology [8,9].

SLC31A1 is an important copper transporter that affects the absorption of dietary copper in cell membranes [10]. Research studies have shown that SLC31A1 is abnormally expressed in various cancer types, with significant implications for patient outcomes and survival rates [11,12]. For example, SLC31A1 is elevated expression in breast cancer samples and predicts poor prognosis [13,14]. Furthermore, glioma patients exhibit heightened SLC31A1 expression levels, which inversely correlate with key survival metrics including overall survival (OS), progression-free survival, and disease-specific survival. Laboratory investigations have demonstrated that SLC31A1 enhances glioma cell proliferation and migratory capabilities [15]. The role of SLC31A1 in PTC has not been specifically reported. At present, only a few studies on gene chips have reported the role of copper cell death in thyroid cancer [16–18]. However, none of these studies addressed copper-related gene expression patterns and their clinical relevance in thyroid cancer. Furthermore, the specific role of SLC31A1 and its relationship with clinicopathological features still need to be further explored in PTC.

This investigation aimed to analyze serum SLC31A1 expression in PTC patients and explore the correlation between it and clinicopathological indicators. At the same time, the effect of SLC31A1 expression level on patient prognosis was evaluated to provide a new perspective for understanding the role of copper death pathway in thyroid papillary carcinoma and explore potential diagnosis and treatment strategies, thereby advancing both our fundamental understanding of PTC biology and the development of targeted treatment strategies.

## 2 Materials and methods

### 2.1 Research object

This study analyzes patients who underwent thyroid surgery at our hospital from January 2017 to August 2021. Among 288 pathologically confirmed PTC cases, 30 individuals did not meet the inclusion criteria and 8 were excluded due to incomplete data, and 250 patients with PTC were finally included as research objects. Participants were divided into good prognosis group (*n* = 205) and poor prognosis group (*n* = 45). All subjects provided written informed consent after being thoroughly informed about the study objectives. This research protocol was approved by our hospital’s Ethics Committee in compliance with the Declaration of Helsinki principles.

### 2.2 Inclusion criteria

Inclusion criteria include: (1) age ≥ 18-years old; (2) first unilateral thyroidectomy or total thyroidectomy; (3) complete clinical data; (4) thyroid ultrasound examination was performed before surgery, and PTC was confirmed by pathology after surgery; and (5) the patient or his family is informed and signs the consent form.

### 2.3 Exclusion criteria

Exclusion criteria include: (1) patients with thyroid disease or taking thyroid-related drugs within the past 3 months; (2) combined with malignant tumors in other parts or serious damage to the heart, brain, liver, kidney, and other vital organs; (3) those who received any antitumor therapy before admission; (4) pregnant or lactating women; and (5) incomplete clinical data.

### 2.4 Collect data

Baseline clinical and pathological data such as age, sex, tumor diameter, degree of differentiation (high, medium, low, and undifferentiated), lesions (single, multiple), TNM stage (stage I–II, stage III–ⅠV), thyroid exocapsular invasion, and lymph node metastasis were collected. About 5 mL of fasting elbow venous blood was drawn from each participants during morning admission and natural agglutination at room temperature for 30 min in the collection tube. After blood coagulation, the upper serum was collected and frozen at −80°C for later use after centrifugation at 2,000 rpm for 20 min.

### 2.5 Enzyme-linked immunosorbent assay (ELISA)

Serum SLC31A1 levels were determined according to ELISA kit instructions. Among them, the SLC31A1 ELISA kit (ABIN6970338) was purchased from antibodies online. All operations were performed in strict accordance with the instructions. Detection range was 0.781–50 ng/mL.

### 2.6 Follow-up and prognosis assessment

Post-discharge follow-up was conducted at 6-month intervals for a period of 3 years, through both outpatient visits and telephone consultations, with the final follow-up date set as August 2024. The 3-year disease-free survival (DFS) rate was calculated for all patients. Poor prognosis was defined as the occurrence of disease recurrence, metastasis, or death during the follow-up period.

### 2.7 Statistical analysis

Statistical analysis and mapping were performed using GraphPad Prism 8.01 Software (GraphPad Software Inc., USA) and SPSS 21.0 statistical software (SPSS, Inc., USA). Data normality was assessed using the Shapiro–Wilk test. Normally distributed continuous variables were expressed as mean ± standard deviation and compared using independent-samples *t*-tests. Categorical variables were presented as frequencies and percentages, with comparisons made using chi-square tests. Survival analysis was conducted using Kaplan–Meier curves, with differences assessed by the log-rank test. Prognostic factors were identified through Cox regression analysis. The predictive value of serum SLC31A1 for poor prognosis was evaluated using receiver operating characteristic (ROC) curves. All statistical tests were two-tailed, with *P* < 0.05 considered statistically significant.


**Informed consent:** All patients involved signed the informed consent form.
**Ethical approval:** This study was reviewed and approved by the Ethics Committee of Hunan Provincial People’s Hospital in compliance with the Declaration of Helsinki principles.

## 3 Results

### 3.1 Clinical baseline characteristics of the enrolled population

This study included 250 patients with PTC as research objects, and divided them into good (*n* = 205) and poor (*n* = 45) prognosis groups based on clinical outcome. Comparative analysis of baselines characteristics (Table 1) revealed no significant differences between two groups in age, gender, BMI, and tumor diameter (all *P* > 0.05). However, significant differences were observed in tumor differentiation degree, lesion status, TNM stage, thyroid extracapsular invasion, and lymph node metastasis (all *P* < 0.05).

**Table 1:** Clinical baseline data

	Good prognosis group (*n* = 205)	Poor prognosis group (*n* = 45)	*P*
**Age (years)**	52.55 ± 7.88	52.91 ± 8.29	0.784
**Gender (** ***n*** **, %)**			
Female	151 (73.66%)	31 (68.89%)	0.579
Male	54 (26.34%)	14 (31.11%)	
**Tumor diameter (cm)**			
<2	75 (36.59%)	15 (33.33%)	0.734
≥2	130 (63.41%)	30 (66.67%)	
**Degree of differentiation (** ***n*** **, %)**			
High and medium differentiation	138 (67.32%)	19 (42.22%)	**0.002**
Low, undifferentiated	67 (32.68%)	26 (57.78%)	
**Lesion status (** ***n*** **, %)**			
Single	120 (58.54%)	18 (40.00%)	**0.031**
Multiple	85 (41.46%)	27 (60.00%)	
**TNM staging (** ***n*** **, %)**			
Stage I–II	128 (62.44%)	20 (44.44%)	**0.030**
Stage III–IV	77 (37.56%)	25 (55.56%)	
**Thyroid exocapsular invasion (** ***n*** **, %)**			
Yes	65 (31.71%)	24 (53.33%)	**0.010**
No	140 (68.29%)	21 (46.67%)	
**Lymph node metastasis (** ***n*** **, %)**			
Yes	60 (29.27%)	24 (53.33%)	**0.003**
No	145 (70.73%)	21 (46.67%)	

*P* < 0.05 indicates a statistically significant difference.

### 3.2 Serum SLC31A1 levels were significantly higher in patients with poor prognosis

**Figure 1:**
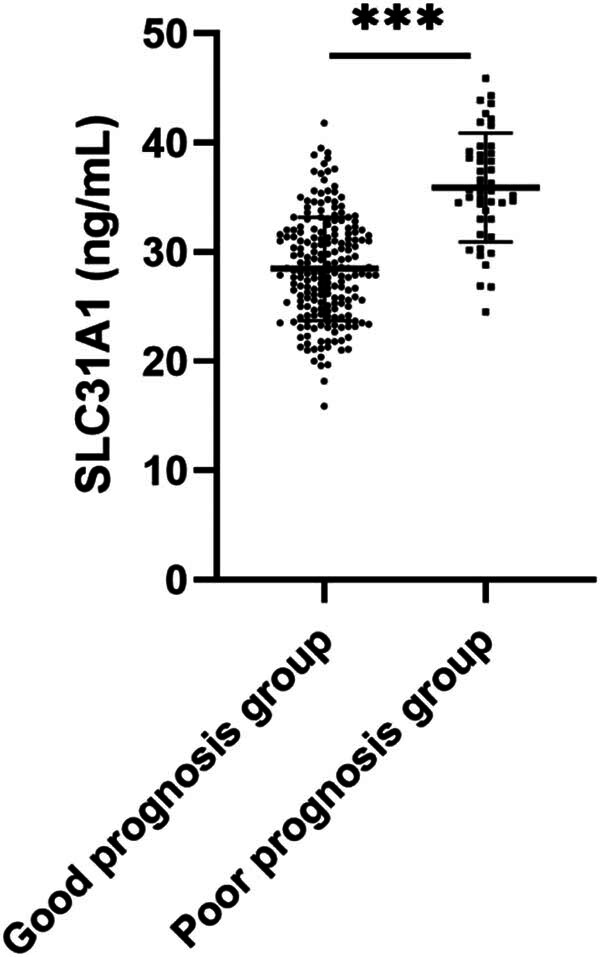
Comparison of serum SLC31A1 levels between good and poor prognosis groups. Serum SLC31A1 levels were determined by ELISA. *** indicates *P* < 0.001.

While SLC31A1 dysregulation has been documented in various malignancies and linked to patient outcomes [11,12], its role in PTC remains largely unexplored. Therefore, we detected the serum SLC31A1 level of PTC patients by ELISA kit, and the results revealed significantly elevated serum SLC31A1 levels in patients with poor prognosis compared to those with good prognosis (*P* < 0.001) (Figure 1), suggesting a potential association between SLC31A1 expression and PTC progression.

### 3.3 Relationship between serum SLC31A1 level and clinicopathological indexes and prognosis in PTC

To investigate association between serum SLC31A1 levels and clinicopathological indicators, PTC patients were stratified into low (*n* = 125) and high (*n* = 205) expression groups based on the median value of serum SLC31A1 value in PTC patients (29.65). Comparative analysis showed no significant differences in age, gender, tumor diameter, and lesion status (all *P* > 0.05) (Table 2). However, results demonstrated high proportions of lower differentiation, advanced TNM stage, and higher percentages of thyroid extranuclear invasion, lymph node metastasis, and poor prognosis (*P* < 0.05). These results indicate that the serum SLC31A1 level in PTC patients is related to differentiation degree, TNM stage, thyroid extranuclear invasion, lymph node metastasis, and prognosis.

**Table 2:** Relationship between serum SLC31A1 level and clinicopathological indexes and prognosis in PTC

	Low SLC31A1 (*N* = 125)	High SLC31A1 (*N* = 125)	*P*
**Age (years)**	52.04 ± 7.71	53.19 ± 8.16	0.252
**Gender (** ***n*** **, %)**			
Female	90 (72.00%)	92 (73.60%)	0.887
Male	35 (28.00%)	33 (26.40%)	
**Tumor diameter (cm)**			
<2	44 (35.20%)	46 (36.80%)	0.895
≥2	81 (64.80%)	79 (63.20%)	
**Degree of differentiation (** ***n*** **, %)**			
High and medium differentiation	90 (72.00%)	67 (53.60%)	**0.004**
Low, undifferentiated	35 (28.00%)	58 (46.40%)	
**Lesion status (** ***n*** **, %)**			
Single	75 (60.00%)	63 (50.40%)	0.162
Multiple	50 (40.00%)	62 (49.60%)	
**TNM staging (** ***n*** **, %)**			
Stage I–II	83 (66.40%)	65 (52.00%)	**0.029**
Stage III–IV	42 (33.60%)	60 (48.00%)	
**Thyroid exocapsular invasion (** ***n*** **, %)**			
Yes	36 (28.80%)	53 (42.40%)	**0.034**
No	89 (71.20%)	72 (57.60%)	
**Lymph node metastasis (** ***n*** **, %)**			
Yes	30 (24.00%)	54 (43.20%)	**0.002**
No	95 (76.00%)	71 (56.80%)	
**Prognosis (** ***n*** **, %)**			
Good prognosis	121 (96.80%)	84 (67.20%)	**<0.001**
Poor prognosis	4 (3.20%)	41 (32.80%)	

*P* < 0.05 indicates a statistically significant difference.

### 3.4 Elevated SLC31A1 expression predicts poor prognosis in PTC

**Figure 2:**
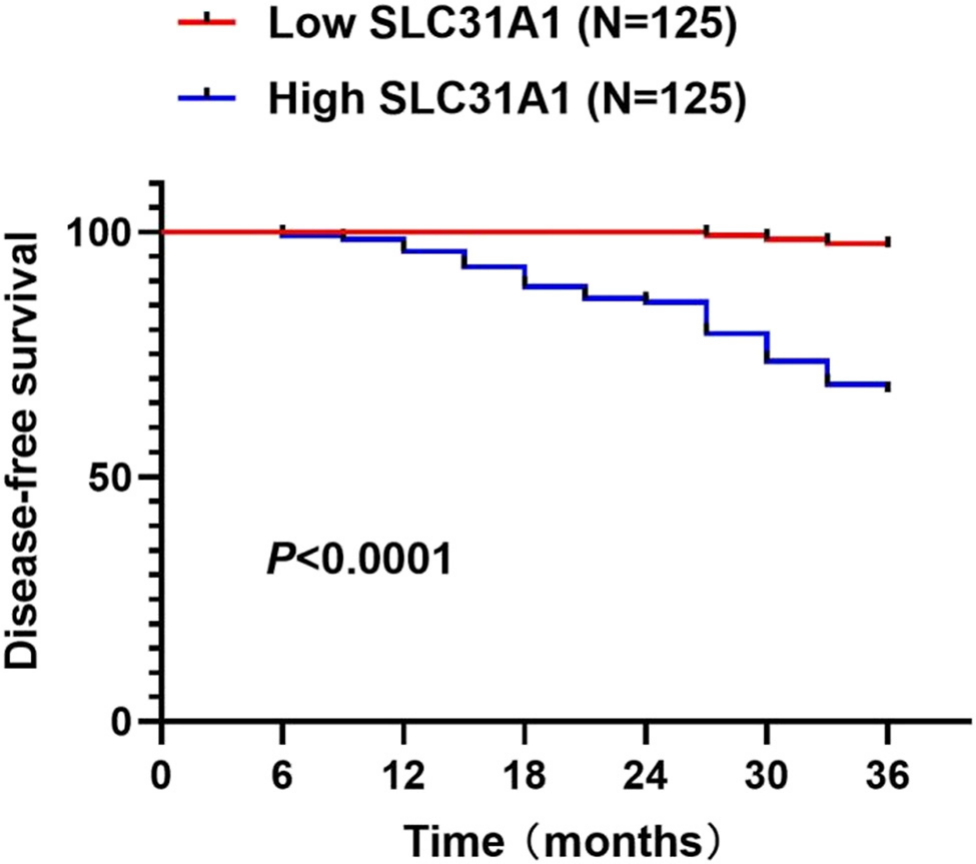
DFS according to SLC31A1 expression levels in PTC.

Kaplan–Meier analysis with log-rank test revealed significantly reduced DFS in patients with high SLC31A1 expression compared to those with low expression (*P* < 0.0001) (Figure 2), suggesting that elevated SLC31A1 levels are associated with adverse outcomes in PTC.

### 3.5 Cox regression analysis of prognostic factors in PTC

Follow-up time was taken as the time variable, and the prognosis of PTC patients was the dependent variable (good = 0, poor = 1). Age, sex, tumor diameter, degree of differentiation, lesions, TNM stage, thyroid extranuclear invasion, lymph node metastasis, and serum SLC31A1 level of the enrolled population in Table 1 were included as independent variables. Univariate analysis showed that the degree of differentiation, lesion status, TNM stage, thyroid epidural invasion, lymph node metastasis, and serum SLC31A1 level were significantly prognostic factors (all *P* < 0.05, Table 3). Subsequent multivariate analysis revealed that poor differentiation, advanced TNM stage, lymph node involvement, and elevated serum SLC31A1 levels were independent predictors of poor prognosis in PTC patients (all *P* < 0.05, Table 3).

**Table 3:** Cox regression analysis of prognostic factors in PTC

Variable	Univariable		Multivariable	
	HR (95% CI)	*P*	HR (95% CI)	*P*
Age (years)	1.006 (0.969–1.044)	0.763	—	—
Gender (*n*, %)	1.231 (0.655–2.314)	0.519	—	—
Tumor diameter (cm)	1.118 (0.602–2.078)	0.724	—	—
Degree of differentiation (*n*, %)	2.627 (1.454–4.748)	**0.001**	1.935 (1.051–3.562)	**0.034**
Lesion condition (*n*, %)	1.991 (1.097–3.615)	**0.024**	1.433 (0.763–2.690)	0.263
TNM staging (*n*, %)	1.923 (1.068–3.463)	**0.029**	1.869 (1.022–3.418)	**0.042**
Thyroid exocapsular invasion (*n*, %)	2.202 (1.226–3.956)	**0.008**	1.163 (0.630–2.148)	0.629
Lymph node metastasis (*n*, %)	2.498 (1.391–4.489)	**0.002**	1.882 (1.039–3.410)	**0.037**
SLC31A1 (ng/mL)	1.266 (1.197–1.340)	**0.000**	1.235 (1.158–1.317)	**0.000**

*P* < 0.05 indicates a statistically significant difference.

### 3.6 Prognostic value of serum SLC31A1 in papillary thyroid carcinoma

**Figure 3:**
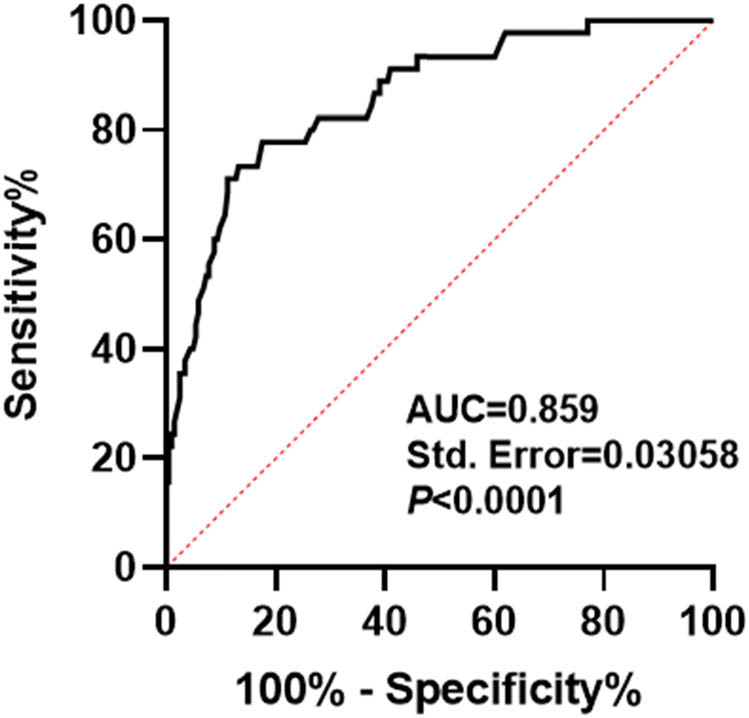
Value analysis of serum SLC31A1 in predicting poor prognosis of PTC patients.

Based on the above results, ROC curve analysis was performed to further assess the prognostic value of serum SLC31A1 in PTC patients. The results showed that the area under ROC curve of serum SLC31A1 level in predicting poor prognosis of PTC patients was 0.859, the sensitivity was 77.78%, and the specificity was 82.44% (Figure 3). These results indicated demonstrating the robust predictive value of serum SLC31A1 for PTC outcomes.

## 4 Discussion

Although PTC is generally considered an inert tumor with a relatively low disease-specific mortality rate, the incidence of local recurrence and distant metastasis is still high, which has a significant impact on patient survival [19,20]. Therefore, the identification of biomarkers related to clinicopathological features and prognosis is of vital significance for optimizing treatment strategies and improving patient prognosis.

SLC31A1 is a major copper transporter, and its high expression may lead to the increase of intracellular copper ion levels, which may affect multiple biological processes, including oxidative stress, DNA damage repair, and apoptosis [11,21]. The elevated intracellular copper ion level caused by high SLC31A1 expression may promote the development of PTC through several mechanisms. First, copper ions can trigger oxidative stress by generating excessive reactive oxygen species (ROS), leading to DNA damage and cell death [22]. Second, copper ions can also activate a variety of signaling pathways, such as PI3K/AKT and NF-κB [23,24]. In addition, copper ions can inhibit apoptosis by modulating the expression of apoptosis-related proteins, thus promoting the survival of tumor cells [25].

SLC31A1 has reportedly as a potential biomarker for cancer therapy and has been associated with chemical resistance in specific cancer types [26,27]. Studies have shown that SLC31A1 is significantly upregulated in multiple malignancies, correlating with decreased OS [11]. Our findings of elevated SLC31A1 expression in PTC patients with poor prognosis align with these observations, suggesting its potential utility as a prognostic indicator in PTC.

Analysis of clinicopathological features revealed that elevated SLC31A1 expression significantly correlated with poor differentiation, advanced TNM stage, extrathyroidal extension, and lymph node involvement, suggesting its role in tumor aggressiveness and metastatic potential. These findings suggest that SLC31A1 may influence prognosis by promoting the aggressiveness and metastasis of cancer cells in PTC. Survival analysis demonstrated reduced DFS in patients with high SLC31A1 expression. Multivariate Cox regression confirmed SLC31A1 as an independent prognostic factor, consistent with previous findings of its potential utility as a prognostic biomarker across multiple malignancies [11].

The high expression of SLC31A1 not only indicates a poor prognosis in PTC, but its copper transport function also suggests potential therapeutic value. Although this study did not involve therapeutic validation, multiple preclinical studies across cancer types have provided theoretical support for SLC31A1-targeted strategies. For example, in osteosarcoma, silencing SLC31A1 via siRNA reverses cisplatin resistance by reducing copper ion uptake [27]. Zinc ions can indirectly inhibit copper transport function by competitively binding to the copper ion-binding domain of SLC31A1. This ion competition mechanism has been verified in enterocytes [28]. In addition, ROS-responsive nanoparticles loaded with elesclomol and copper ions can serve as a targeted drug delivery system, enabling specific release of the drugs at SLC31A1 highly expressed tumor sites. This approach enhances antitumor effects by synergistically inducing copper death and immunogenic cell death [9], providing a technical reference for the precise treatment of PTC. Although the aforementioned studies have not been directly validated in PTC, the SLC31A1-dependent copper metabolism mechanism has revealed cross-cancer commonality. Subsequent research may focus on the following directions: in PTC cell lines (such as TPC-1, K1), SLC31A1 perform knockdown or overexpression to detect intracellular copper ion concentration, ROS levels, phosphorylation status of signaling pathways, and expression of apoptosis-related proteins (such as Bcl-2, Bax); reverse phenotypes using copper chelators (such as tetrathiomolybdate) to confirm copper metabolism dependency; construct SLC31A1-overexpressing xenograft tumor models in nude mice, and use combined SLC31A1 inhibitors with copper carriers like elesclomol to observe changes in tumor growth, metastasis, and apoptotic markers (such as Cleaved caspase-3); use immunoprecipitation to screen SLC31A1-interacting proteins (such as ATP7A) to clarify copper transport mechanisms, and employ RNA-seq technology to mine downstream antioxidant stress genes and epithelial–mesenchymal transition-related pathways. The above research will provide key experimental evidence for the transformation of SLC31A1 from a prognostic marker to a therapeutic target, promoting the development of individualized treatments for PTC.

ROC analysis demonstrated the robust prognostic utility of serum SLC31A1 in PTC. This finding provides clinicians with a new biomarker to help identify high-risk patients early and treat them accordingly. However, it is important to note that the predictive power of a single biomarker is limited, and future studies may consider combining other biomarkers or clinical indicators to improve the accuracy of predictions.

The detection of serum SLC31A1 holds multidimensional application potential in the clinical management of PTC. Specifically, serum SLC31A1 levels could serve as a non-invasive biomarker for postoperative risk stratification, allowing clinicians to identify high-risk patients (e.g., those with advanced TNM stage or poor differentiation) who may benefit from intensified surveillance or adjuvant therapies. Additionally, serial monitoring of SLC31A1 levels could aid in early detection of recurrence by reflecting tumor burden changes, complementing traditional imaging techniques. For treatment selection, high SLC31A1 expression may predict sensitivity to copper-targeted therapies (e.g., elesclomol) or inform combination strategies with other molecularly targeted agents (e.g., BRAF inhibitors in V600E-mutated tumors). Furthermore, integrating SLC31A1 into existing prognostic models (such as MACIS or AMES) could enhance their accuracy in predicting long-term outcomes. While these applications require validation in larger, multicenter cohorts, this study provides a foundational rationale for exploring SLC31A1 as a tool to guide personalized management of PTC patients.

In summary, the high expression of SLC31A1 in thyroid papillary carcinoma is associated to poor clinicopathological features and prognosis, and SLC31A1 may become a potential therapeutic target for PTC. Although this study provides important information about SLC31A1 in PTC, there are some limitations. The single-center design and relatively small sample size may limit result generalizability. Additionally, this study only discussed the relationship between the expression of SLC31A1 and clinicopathological indicators and prognosis, without further exploring its specific molecular mechanism. Future studies could further validate the mechanism of action of SLC31A1 in PTC through *in vitro* experiments and animal models, and explore its possibility as a potential therapeutic target.
